# A web- and mobile phone-based intervention to prevent obesity in 4-year-olds (MINISTOP): a population-based randomized controlled trial

**DOI:** 10.1186/s12889-015-1444-8

**Published:** 2015-02-07

**Authors:** Christine Delisle, Sven Sandin, Elisabet Forsum, Hanna Henriksson, Ylva Trolle-Lagerros, Christel Larsson, Ralph Maddison, Francisco B Ortega, Jonatan R Ruiz, Kristin Silfvernagel, Toomas Timpka, Marie Löf

**Affiliations:** Department of Biosciences and Nutrition, Karolinska Institutet, NOVUM, Huddinge, 141 83 Sweden; Department of Medical Epidemiology and Biostatistics, Karolinska Institutet, Stockholm, PO 281, 171 77 Sweden; Department of Clinical and Experimental Medicine, Faculty of Health Science, Linköping University, Linköping, 581 85 Sweden; Clinical Epidemiology Unit, Department of Medicine, Karolinska Institutet, Stockholm, PO 281, 171 77 Sweden; Department of Food and Nutrition and Sport Science, University of Gothenburg, Gothenburg, PO 100, 405 30 Sweden; National Institute for Health Innovation, The University of Auckland, Auckland, PO 92019, 1142 New Zealand; PROmoting FITness and Health through physical activity research group (PROFITH), Department of Physical Education and Sports, Faculty of Sport Sciences, University of Granada, Granada, 18071 Spain; Department of Behavioral Sciences and Learning, Linköping University, Linköping, 581 83 Sweden; Department of Medical and Health Sciences, Faculty of Health Science, Linköping University, Linköping, 581 85 Sweden

**Keywords:** Childhood obesity, Randomized controlled trial, Mobile phones, Body composition

## Abstract

**Background:**

Childhood obesity is an increasing health problem globally. Overweight and obesity may be established as early as 2–5 years of age, highlighting the need for evidence-based effective prevention and treatment programs early in life. In adults, mobile phone based interventions for weight management (mHealth) have demonstrated positive effects on body mass, however, their use in child populations has yet to be examined. The aim of this paper is to report the study design and methodology of the MINSTOP (Mobile-based Intervention Intended to Stop Obesity in Preschoolers) trial.

**Methods/Design:**

A two-arm, parallel design randomized controlled trial in 300 healthy Swedish 4-year-olds is conducted. After baseline measures, parents are allocated to either an intervention- or control group. The 6- month mHealth intervention consists of a web-based application (the MINSTOP app) to help parents promote healthy eating and physical activity in children. MINISTOP is based on the Social Cognitive Theory and involves the delivery of a comprehensive, personalized program of information and text messages based on existing guidelines for a healthy diet and active lifestyle in pre-school children. Parents also register physical activity and intakes of candy, soft drinks, vegetables as well as fruits of their child and receive feedback through the application. Primary outcomes include body fatness and energy intake, while secondary outcomes are time spent in sedentary, moderate, and vigorous physical activity, physical fitness and intakes of fruits and vegetables, snacks, soft drinks and candy. Food and energy intake (Tool for Energy balance in Children, TECH), body fatness (pediatric option for BodPod), physical activity (Actigraph wGT3x-BT) and physical fitness (the PREFIT battery of five fitness tests) are measured at baseline, after the intervention (six months after baseline) and at follow-up (12 months after baseline).

**Discussion:**

This novel study will evaluate the effectiveness of a mHealth program for mitigating gain in body fatness among 4-year-old children. If the intervention proves effective it has great potential to be implemented in child-health care to counteract childhood overweight and obesity.

**Trial registration:**

ClinicalTrials.gov NCT02021786; 20 Dec 2013.

## Background

According to the World Health Organization, childhood obesity is one of the most serious public health challenges of the 21st century [1]. Evidence-based prevention and treatment programs are required in order to counteract this increased prevalence of childhood overweight and obesity. An important consideration is at what time period in life such actions ought to be initiated. Previously, a high body weight in relation to height was not considered to be a problem in pre-school children (2–5 years) [2], however, this point of view is now challenged [2,3]. Children who are overweight during their pre-school years tend to remain overweight throughout childhood and adolescence [4-6]. Thus, development of treatment and preventive interventions addressing overweight and obesity are justified among young children [7,8]. Despite this, the evidence base for effective interventions in this age group is still very limited [8-10].

For behavioral treatment of obesity interventions, face-to-face sessions have traditionally been the preferred mode of delivery. Such approaches are expensive, difficult to scale up, and require transportation to hospitals or primary health care units. In adults, the use of programs based on mobile phone technology for delivering behavior change interventions (mHealth) has attracted increased research interest e.g. [11,12]. The benefits of mHealth interventions include that they can be delivered anywhere at any time, participants are not required to attend a clinic and they provide opportunities for interactivity and tailoring to specific groups. Several reviews have also concluded that mHealth programs may be useful for achieving changes in behavior and weight loss [13-16]. However, no study to date has evaluated a mHealth solution to counteract overweight and obesity in pre-school children.

In intervention studies, the use of an appropriate measure to identify overweight and obesity is important. Previous studies have commonly used BMI e.g. [17-20], however this does not provide any indication of body fatness in young children [21]. Variations in BMI have been reported to only explain 15% of the variation in body fatness in 4-year-olds [22]. Until recently, simple and accurate estimates of body fatness have been difficult to obtain. However, equipment based on the principle of air-displacement plethysmography [23], which for some time has been available for adults and infants, also became available for 2 to 6 year olds in 2011 [24]. In a recent publication using this equipment, we reported that healthy Swedish 4-year-olds contained on average 25.2 (boys) and 26.8 (girls) percent body fat, and this figure is in fact almost 60% higher than the reference values for body composition at this age presented by Fomon et al. [25]. Clearly, although cut-off criteria for optimal body fatness in young children have yet to be established, this fact illustrates that preventive strategies to counteract further increase in body fatness in children are motivated.

### Aim

The aim of this paper is to report the study design and methodology of the MINSTOP (Mobile-based Intervention Intended to Stop Obesity in Preschoolers) trial. The overall goal of MINISTOP is to determine the effectiveness of a 6-month mHealth parental intervention on body fatness, dietary habits, physical fitness, physical activity and sedentary behavior in 4-year-olds.

### Hypotheses

The primary hypothesis is that the MINISTOP program will be effective at improving body composition at six months compared to the control group. Secondary hypotheses are that participants in the MINISTOP program will spend more time in moderate to vigorous physical activity and less time in sedentary activities; have improved physical fitness, and consume more fruits and vegetables and less candy and soft drinks.

## Methods

### Study design

MINISTOP is a two-arm parallel design randomized controlled trial. After baseline assessment, participants are randomized to either the intervention or the control group in a 1:1 ratio. The intervention group receives a program delivered through a software application for smartphones (the MINISTOP app), whereas the control group receives information/advice about a healthy diet and physical activity via a four-page pamphlet. The information is based on current guidelines [26] and corresponds to the information provided by the Swedish child health care system. The protocol is in accord with the SPIRIT 2013 statement [27,28] and the intervention is described according to the CONSORT –EHEALTH checklist (v1.6.1) [29].

### Eligibility and recruitment

To be included in MINISTOP, parents must: have a four year old child and live in the county of Östergötland (population 435 000), Sweden; have the possibility to have their child measured at baseline at 4.5 years ± 2 months of age; and be able to speak and read Swedish sufficiently well (at least one parent). Children diagnosed with neurological or endocrine diseases and children who have a parent suffering from a serious physical or psychological disease making the study too demanding for the family are excluded from the study.

**Figure 1:**
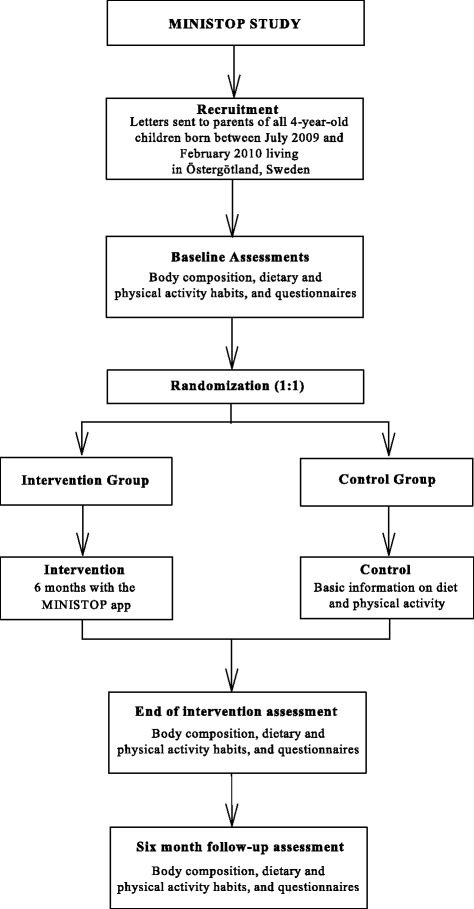
**Flow chart of the MINISTOP study design.**

Participants are recruited from a population-based sample drawn from the population register at Statistics of Sweden. The primary sample consists of all 4-year-olds (born between July 2009 and February 2010) living in the county of Östergötland and include approximately 3000 children. Invitation letters are sent out to the home addresses of the guardians (parents and other caretakers) asking them to participate. Parents/caretakers that consent to participate bring their child to the hospital for a baseline assessment of their child’s body fatness and physical fitness. Thereafter, the child’s baseline physical activity and diet are assessed during the subsequent two-week period. When all the baseline measures are completed, the children are randomized either to the intervention or the control group. All outcome measures (body fatness, dietary variables, physical fitness and physical activity variables) are repeated after the intervention (six months after baseline) and at follow-up (twelve months after baseline). Figure 1 outlines the study design.

### Randomization and blinding

After the baseline assessment is completed, the participants are randomly allocated to either the control (no intervention) or intervention (MINISTOP) arms at 1:1 ratio by means of a random allocation sequence list (in blocks) generated using SAS 9.4 (SAS Institute Inc, Cary NC). The allocation sequence is overseen by the project statistician (SS). Assessors of the primary outcomes are blinded to treatment allocation; however, due to the nature of the intervention participants are not blind to their allocation.

### Intervention

#### Overall

The overall goal of MINISTOP is to have the pre-school children achieve healthy body fatness through increasing physical activity and improving dietary behavior. The MINISTOP intervention consists of a 6-month program delivered via a mobile web-application (app), which is compatible for both iOS (version 6.1.3 or higher) and Android (version 2.3.5 or higher) smartphones.

#### Development

The MINISTOP app was developed by a team of researchers with expertise in nutrition, behavioral science, medicine, engineering, psychology and physical activity. Intervention content is grounded in the Social Cognitive Theory [30] and includes evidence-based recommendations for obesity interventions targeting young children [31]. Content was first tested in a pilot study with 10 parents of a 4-year-old child recruited from day care centers in the county of Östergötland in October 2013. After reiteration of the intervention based on parents’ comments (obtained by means of a questionnaire), an additional 19 parents with a 4-year-old child were recruited and user tested a demonstration version of the MINISTOP app in December 2013. They provided comments on both content and feasibility by means of a questionnaire. These comments were used for the final modifications of the app before the MINISTOP trial was initiated.

#### Content and use

MINISTOP involves the delivery of a comprehensive program of information and push notifications based on existing guidelines for a healthy diet and activity in pre-school children [26]. It covers twelve topics that change every other week. Topic themes include healthy foods in general, breakfast, healthy small meals, physical activity and sedentary behavior, candy and sweets, fruits and vegetables, drinks, eating between meals, fast food, sleep, foods outside the home, and foods at special occasions. Every second week the parents are alerted via a push notification within the application that a new topic has begun. For each topic, general information, tips and strategies for how to change an unwanted behavior are provided. Using the app, and according to personal preference, parents can record their child’s intake of fruits and vegetables, sugar sweetened beverages and candy as well as physical activity and sedentary behavior. At the end of each week parents’ responses are summarized and graphically presented for the parents in their mobile phones in order to increase motivation and compliance. The app also provides written feedback based on these graphs at three different levels by means of a large number of automated messages. Throughout the intervention period, the parents are offered the possibility to interact on a regular basis by means of the app with a dietician and a psychologist in order to discuss problems and raise questions regarding the promotion of a healthy diet and physical activity for their child. If parents have not used the app for a couple of days, they receive a reminder message via the app at 8 pm (maximum four times a week).

#### Download

At the beginning of the intervention, parents receive a username and password to the MINISTOP app by email as well as a text message (SMS) with a website link. They are asked to download the app as soon as possible by clicking the website link. After two days, one of the investigators contacts the parent and provides instructions on how to use the app. For parents that are living together, both parents are asked to download the app to their mobile phones (using the same username and password). Both are instructed to read all information, however, only one should register the child’s behavior throughout the intervention period. For parents that are separated, and when the child lives at two places (e.g. one week at each place), both parents are instructed to download the app (with different user names). Both are then instructed to record their child’s behaviors (when the child is living with them).

### Sample size and power considerations

The MINISTOP trial was carefully dimensioned beforehand in order to provide sufficient power to assess if the intervention is successful. We aim to recruit 300 children to ascertain that 200 will complete the follow-up measures. A total of 200 children (100 in each group) completing the study will provide sufficient statistical power (>80%) at the two-sided 5% significance level to detect a difference of 10% (log 1.1 = 0.095) in energy intake and body fatness for the intervention group compared with the control group over the study interval of 6 months. In our calculations we assumed three levels of standard deviations in energy intake and body fatness (log values: 0.18, 0.22 and 0.24, respectively).

## Measures

Primary outcomes are body fatness and energy intake, while secondary outcomes include time spent in sedentary, moderate, and vigorous physical activity, physical fitness and intakes of fruits and vegetables, snacks, soft drinks and candy.

### Body composition

Body fatness is assessed using air-displacement plethysmography by means of the pediatric option for BodPod (http://www.cosmed.com) as previously described [22]. The pediatric option measures body volume and body density is then calculated as body volume divided by weight. Body density is then converted into body fatness using appropriate densities of fat and fat-free mass [32]. The pediatric option for BodPod is able to provide accurate and precise estimates of fat and fat-free mass in children aged 2–6 years [24].

### Food and energy intake

Intake of foods and drinks and dietary energy are assessed using our newly developed procedure (Tool for Energy balance in Children, TECH). Using this approach, parents and other caretakers photograph all food items and drinks consumed by the child using a mobile phone camera during 4 × 24-hour-periods. All foods and drinks are photographed from two different angles together with a marker of known size. The parents are also given standardized china (plate, bowl and cup) to allow for more accurate food and drink calculations. The parents also provide a short description of the food and drink the children has consumed throughout the four-day period. Portion sizes of foods and drinks are estimated from the photographs and analyzed by trained nutritionists and dieticians. The intake of foods and drinks are then converted to energy intake through linkage the Swedish Food Database, National Food Administration [26]. In a pilot study we have shown that mean intakes of energy as well as fruits and berries, vegetables, and juice were in agreement with total energy expenditure using the doubly labelled water method and corresponding intakes using a food frequency questionnaire (Henriksson et al., submitted). The accuracy of TECH will also be evaluated within the MINISTOP trial in forty children (20 in the intervention group and 20 in the control group). In these children, energy intake obtained using TECH will be compared to total energy expenditure measured using the doubly labelled water method as previously described [33]. Intake of energy, foods and drinks using TECH will also be compared to corresponding estimates with telephone 4×24-hours-recalls covering the same time period. The telephone interviews will be conducted and evaluated by a trained dietician using the Swedish Food Database [26].

### Physical activity and sedentary behavior

Physical activity and sedentary behavior are assessed over seven consecutive days using the Actigraph wGT3x-BT triaxial accelerometer (http://www.actigraphcorp.com), which has previously been shown to be able to provide accurate estimates of activity energy expenditure as well as classify sedentary, light, and moderate/vigorous levels of physical activity in children aged 3–5 years [34]. Parents and other caretakers are asked to record when the device has been taken off and the activities performed during that period (i.e. showering/bathing/swimming) in a notebook. Data acquisition storage is set in 15 second-epochs.

### Physical fitness

Physical fitness will be assessed using the PREFIT fitness battery which includes the following five fitness tests [35]: the handgrip strength test (for upper musculoskeletal fitness), the standing long jump test (for lower musculoskeletal fitness), the 4×10 m shuttle run test (for motor fitness), the one-leg stance test (for motor fitness) and the 20 m shuttle run test (for cardiorespiratory fitness). Details on how these tests are performed have been published previously [35]. All the tests are conducted twice and the best score is retained, except for the one-leg stance test and the 20 m shuttle run test that are performed only once.

### Other measures: demographic and health-related behaviors

At baseline, parents complete demographic questions (age, sex and socio-economic status), as well as answer questions regarding their child’s health and their own body weight, height, dietary and exercise habits, education, smoking habits, health, and medications. Parents also fill in a questionnaire regarding parental self-efficacy for promoting healthy physical activity and dietary behaviors in children [36], the Swedish Parenthood Stress Questionnaire (SPSQ) [37] and the Strengths and Difficulties Questionnaire (SDQ) [38] at baseline, at the end of the intervention and at follow-up. Finally, online food frequency questionnaires and physical activity questionnaires [39-41] are sent via email to each of the parents and the child (to be filled out by one of the parents) to assess the dietary and physical activity habits of the families participating.

## Statistical analyses

Our project statistician (SS) has together with the research team outlined a detailed analytic stepwise strategy to evaluate if the intervention is successful or not. The details of this strategy will be published together with the results. Briefly, the steps include both an evaluation on the group level for primary as well as secondary outcomes, and summary scores where a combination of both primary and secondary outcomes is used. For primary and secondary outcomes, we will compare intervention and control by testing the hypothesis that the two groups have equal effect in location or distribution versus the hypothesis that the two groups differ. This will be done by calculating the stratified Wilcoxon rank sum test (continuous variables such as body fat). Variables on a binary scale will be compared by fitting exact logistic regression. We will apply intention to treat analysis. Finally, all analyses will be made stratified by baseline body fatness (tertiles). This is done since children can be expected to respond and develop differently depending on their baseline status. A child with a high body fat content with a sedentary behavior and unhealthy eating habits will have larger potential for improvement while a child with lower body fatness and healthier eating and activity habits will have less potential. All statistical analyses will be conducted using SAS version 9.4 (SAS Institute Inc, Cary NC) using the two-sided 5% level of significance.

## Ethics approval

This trial was approved by the Research Ethics Committee, Stockholm, Sweden (2013/1607–31/5; 2013/2250–32). Verbal informed consent, witnessed and formally recorded, is obtained from the parents.

## Trial status

The recruitment for the MINISTOP trial was initiated in the spring of 2014. The project is progressing in accordance with the time plan. The baseline measures were completed in October 2014. The measures after the intervention (six months after baseline) are on-going and will be completed in April 2015. The follow-up measures 12 months after the baseline measures will be completed in October 2015.

## Discussion

The MINISTOP trial will evaluate the impact of a mHealth intervention to counteract obesity in 4-year-old children. Worldwide, very few interventions have been conducted in children under 5 years of age [8,9,42], and none to date have evaluated mHealth interventions.

Our trial has several strengths, including the randomized controlled trial design, careful calculation of statistical power, and objective assessment of the primary outcome body composition using a reliable and valid method. In addition, participating children are recruited from a population-based sample drawn by Statistics Sweden. Finally, the intervention is grounded in behavior change theory, was developed in accordance with the mHealth development framework [43], and strategies for promoting behavior change are evidence based. The MINISTOP trial is limited by the fact that the intervention content is in the Swedish language, thus it only includes parent couples where at least one parent speaks and reads Swedish sufficiently well.

Some technical aspects of our mHealth intervention should be acknowledged. We use a web-application instead of a native app which has several technical advantages. Firstly, it allows us to include both Android and iOS smartphone users, which increases its feasibility and user-friendliness. Furthermore, this solution enables us to collect and analyze data on usage of the app throughout the intervention, and yet few studies have done this to determine adherence and fidelity of mHealth interventions. Finally, the app only targets the parents since this trial includes very young children. However, subsequent iterations might include features targeting the children themselves, such as gamification to increase intake of vegetables [44].

We chose our study design with a general recruitment since it is robust against unforeseen changes as well as against assumptions about the actual data distributions and function of the intervention. An alternative design could have been to include only children with a BMI above a certain cut-off, however, BMI is less accurate measure of body fatness and furthermore it is not necessarily a true assumption that the best success will be among children with extreme levels of BMI or body fatness. The generalizability of this study will also be optimized by a general and unrestricted recruitment. There are also risks associated with the approach since we are also including normal-weight children if the intervention only has effect among children with more extreme values of body fatness our effect will be diluted. In this respect it is also relevant to note that our intervention provides information on healthy eating and active lifestyle that apply to children in general. In summary, we decided to include all children instead of selecting sub-groups to avoid problems with regression to the mean and decrease the problem with selections.

If the MINISTOP app turns out to be successful it has potential to be implemented in child-health care to counteract childhood obesity. This is important since childhood obesity is an independent risk factor for adult obesity [45], which increases the risk for adverse health conditions such as cardiovascular disease and diabetes [46]. If fewer children are overweight and obese, fewer adults will be affected and thus the prevalence of obesity-related disorders will decrease. Such a development will decrease costs for the health care system and improve physical and psychosocial health of individuals.
